# Tactile force evokes a biphasic BOLD response in ipsilateral primary somatosensory cortex

**DOI:** 10.1162/IMAG.a.1236

**Published:** 2026-05-21

**Authors:** Anna C. Feldbush, Nahid Kalantaryardebily, Neha A. Reddy, Rebecca Faubion-Trejo, Jeff Soldate, Jonathan Lisinski, Molly G. Bright, Netta Gurari, Stephen M. LaConte

**Affiliations:** Fralin Biomedical Research Institute at VTC, Virginia Tech, Blacksburg, VA, United States; Neuroscience, Virginia Tech, Blacksburg, VA, United States; Mechanical Engineering, Virginia Tech, Blacksburg, VA, United States; Physical Therapy and Human Movement Sciences, Northwestern University, Chicago, IL, United States; Biomedical Engineering, Virginia Tech, Blacksburg, VA, United States

**Keywords:** negative BOLD, multi-echo fMRI, tactile signaling, primary somatosensory cortex

## Abstract

Tactile perception is fundamental to how we engage with and interpret our surroundings. While the contralateral primary somatosensory cortex (S1) is well known to play a role in processing tactile information, lesser understood responses in the ipsilateral hemisphere have been observed. We aim to characterize the blood-oxygen-level-dependent (BOLD) response pattern of ipsilateral S1 to unilateral tactile stimuli. Three stimulus force levels were applied in an event-related design. As expected, we observed an apparent difference in BOLD responses between the contralateral and ipsilateral hemispheres. We found differing response patterns between the Brodmann’s areas (BAs) within ipsilateral S1. Ipsilateral BA2 had a positive BOLD response similar to that of the contralateral hemisphere. In contrast, ipsilateral BA1 and BA3b appeared to have a biphasic response. That is, those regions had an initial negative response, followed by a secondary positive one. Using multi-echo analysis, we evaluated whether this biphasic response pattern is BOLD related. We hypothesize that this biphasic response is driven by two periods of neural activity. In addition, the secondary (positive) phase was more sensitive to tactile stimulus force level than the initial “negative” BOLD phase. The ipsilateral BA1 and BA3b responses support a previous hypothesis of bilateral sensory gating. This study extends previous research reporting negative responses in ipsilateral S1, suggesting that the response is consistent with a BOLD signal, biphasic, and that the previously overlooked secondary positive phase is actually more sensitive to stimulus intensity.

## 1 Introduction

While it is well known that the contralateral primary somatosensory cortex (S1) responds to unilateral tactile stimuli, our understanding of ipsilateral responses has evolved significantly over the last three decades. Clarifying the functional role of ipsilateral S1 may yield valuable insights into a phenomenon that is both poorly understood and often overlooked. Beyond its implications for interhemispheric integration and distributed neural processing, ipsilateral responses may provide crucial insights into compensatory function after peripheral nerve damage (Fornander et al., 2016) and central neurological injury from stroke and traumatic brain injury. Early electrophysiology studies in neurosurgery patients first suggested that median nerve stimulation could evoke ipsilateral responses (Allison et al., 1989; Noachtar et al., 1997). The occurrence and characterization of ipsilateral S1 responses have since been studied with EEG (Sutherland & Tang, 2006), MEG (Kanno et al., 2003; Korvenoja et al., 1995, 1999; Lipton et al., 2006; Schnitzler et al., 1995), and fMRI (Blatow et al., 2007; Eickhoff et al., 2008; Korvenoja et al., 1999; Nihashi et al., 2005; Stringer et al., 2014). However, ipsilateral findings are less robust and less reproducible than contralateral S1 responses. Moreover, the interpretation of ipsilateral responses in fMRI studies has been complicated by the observation that median nerve stimuli tend to evoke negative blood-oxygen-level-dependent (BOLD) responses in ipsilateral S1 (Blatow et al., 2007; Eickhoff et al., 2008; Hlushchuk & Hari, 2006; Kanno et al., 2003; Klingner, Huonker, et al., 2011; Klingner et al., 2014; Lamp et al., 2019; Lipton et al., 2006; Nihashi et al., 2005; Schäfer et al., 2012; Stringer et al., 2014). Similar to negative BOLD phenomena reported in the visual cortex (Amedi et al., 2005; Shmuel et al., 2006), definitive mechanistic interpretations of negative BOLD in somatosensory cortex remain elusive (Gouws et al., 2014; Klingner et al., 2014; Nelson & Mayhew, 2025; Schäfer et al., 2012).

To address these challenges in interpreting negative responses in ipsilateral S1, the current study focuses on two important facets of BOLD measurements. First, we employed a multi-echo echo-planar imaging (EPI) sequence to assess the echo-time (TE) and T2* dependence of the ipsilateral response. This enabled us to evaluate whether observed ipsilateral signals are indeed related to the BOLD mechanism. Second, we examined the response time course in ipsilateral S1 to explore the possibility of serial processing phases, leveraging the fact that our stimulus duration was brief compared with most previous studies. Early multi-echo work by Peltier and Noll (2002) corroborated resting-state data as neurovascularly coupled (BOLD related) as opposed to arising from non-neuronal artifacts such as vascular symmetry, motion, physiological noise, and scanner instability. Further work has built on echo-time dependence as a hallmark characteristic of the BOLD response (Devi et al., 2022; DuPre et al., 2021; Havlicek et al., 2017; Kundu et al., 2012; N. Li & Jasanoff, 2020). Generally, this approach leverages the TE dependence and decay properties of the signal to validate whether a given fluctuation arises from changes in T2*, which is the fundamental basis of the BOLD contrast. However, with notable exceptions (e.g., Bianciardi et al., 2011), multi-echo experiments have not been broadly applied to the characterization of the negative BOLD response.

Beyond T2* measurements, improved characterization of the ipsilateral S1 response is critical for interpreting the neural and physiological underpinnings of BOLD signals. Within rather broad ranges of stimulus parameters, the BOLD signal can be approximated as the stimulus timing convolved with a hemodynamic response function (HRF) (Glover, 1999; Henson & Friston, 2007), although it is known that distinct neural or vascular mechanisms are not always well captured by canonical HRF models (Chen et al., 2023). Standard HRF modeling assumes that a brief stimulus elicits a brief neural event, which is then convolved with a fixed hemodynamic response. However, this simplified model may be inadequate for capturing the complexity of brain activity. Specifically, a brain region may exhibit a multi-phased neural response that is not accurately reflected by the standard HRF. This mismatch can lead to poor model fit and inaccurate estimation of neural activity. Nevertheless, HRF convolution modeling remains reasonably accurate, even in the cases where these known limitations play a role (Boynton et al., 1996; Vazquez & Noll, 1998).

Importantly, most studies reporting negative BOLD responses in ipsilateral S1 have used block designs. This enhances detection sensitivity since ipsilateral responses are typically weaker and less reliable than contralateral ones. However, upon reviewing this literature, we observed that many of the reported negative responses deviate from the expected shape predicted by standard HRF convolution models (e.g., Hlushchuk & Hari, 2006; Klingner, Huonker, et al., 2011). Specifically, given a block design stimulus of duration S and an HRF of duration H, the expected BOLD response (B) should equal S + H – 1 for sampled, discrete signals. Thus, a negative BOLD HRF under a block-design stimulus should produce a negative signal convolved beyond the duration of the block. Instead, previous reports frequently show negative BOLD responses lasting for only a portion of the stimulus duration (de la Rosa et al., 2021; Hlushchuk & Hari, 2006; Kastrup et al., 2008; Shmuel et al., 2006). We propose three potential explanations for this discrepancy. First is that the decrease in signal after a stimulus is a non-neural vascular artifact. Past work suggests that this is not the case as the negative signal changes in response to a vasodilatory stimulus, like breath holding, have been shown to reflect distinctly different mechanisms from those observed in response to neural stimuli (Bright et al., 2014), and likely not driven by BOLD contrast effects (Bianciardi et al., 2011). If the response is, in fact, neurally driven, we expect to see BOLD weighting. In this case we propose the additional two alternatives. A potential second explanation is that the negative BOLD response fundamentally violates linearity assumptions, suggesting mechanisms independent of neurovascular coupling. A third explanation would be that the response we are capturing reflects two distinct phases of neurovascular activity. Such a biphasic response could account for the short negative signal phase reported in previous studies where the negative response ends before the stimulus block itself concludes. Based on theoretical considerations and preliminary observations, we favored this third biphasic hypothesis, suspecting it had been overlooked in earlier studies, potentially due to their long stimulus durations. To test this explicitly, we used a pneumatically controlled stimulation device capable of delivering robust yet brief (<1 s duration) tactile stimuli to the tip of the right index finger at three distinct force levels. Note that initial analysis of the data in this study was previously reported in Kalantaryardebily et al. (2025) to validate our in-house stimulation delivery system. Given that our stimulus duration was relatively brief (S < H), we predicted that the negative BOLD response would persist beyond the duration of the stimulus, which would not be the case for longer block designs. Informed by foundational studies on negative BOLD in S1 (Blatow et al., 2007; Eickhoff et al., 2008; Hlushchuk & Hari, 2006; Kanno et al., 2003; Klingner, Huonker, et al., 2011; Klingner et al., 2014; Lamp et al., 2019; Lipton et al., 2006; Nihashi et al., 2005; Schäfer et al., 2012; Stringer et al., 2014), we performed region of interest (ROI) analyses specifically targeting ipsilateral S1.

## 2 Methods

### 2.1 Participants

We collected fMRI data from 14 right-hand dominant individuals (male: 7, female: 7), as classified by the Edinburgh Handedness Inventory (Robinson, 2013). Participants were aged 18–23 (21 ± 2 years). Additionally, participants reported no history of neurological or musculoskeletal injuries that could affect tactile signaling. Data collection was approved by Virginia Polytechnic Institute and State University’s Institutional Review Board. All participants provided informed consent before experimentation and were aware of the study design and the nature of the tactile stimuli we provided. Participants were instructed that they could withdraw from the study at any time.

### 2.2 Tactile stimulation

**Fig. 1.**
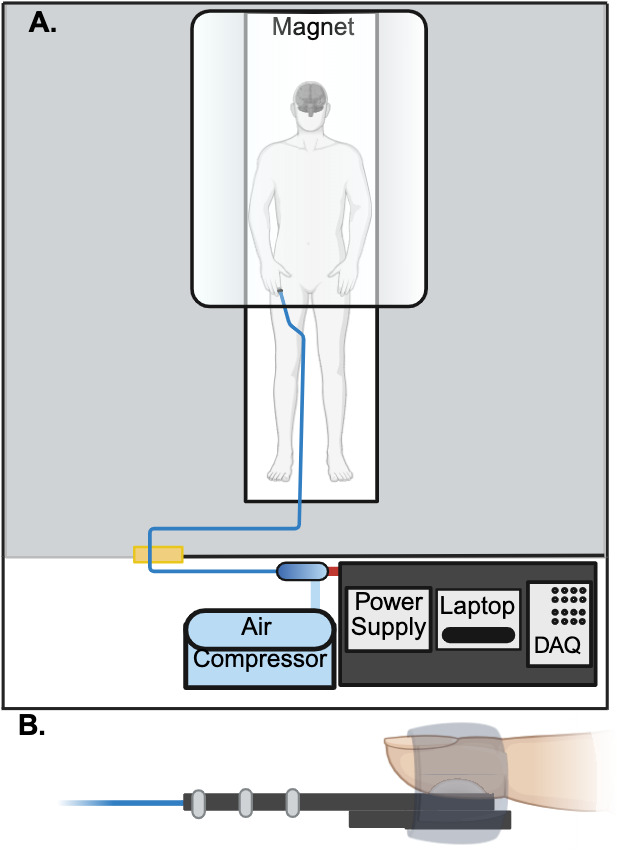
fMRI compatible tactile stimulator. (A) Schematic of the tactile stimulator system we used in this fMRI study. All electronic components were housed in the MRI control room. The pneumatic tubing entered the MRI scanner room via a wave guide. All components within the MRI scanner room were non-ferrous (Kalantaryardebily et al., 2025). (B) View of the tactile stimulator, which was affixed to the participant’s finger using medical tape.

We applied tactile stimuli to the distal phalanx of the right index finger using a custom-designed pneumatically controlled actuator (Fig. 1; Kalantaryardebily et al., 2025). This device provides tactile stimuli by inflating a small-diameter balloon. Our experiment used three different stimulus levels (Low: 88.7 kPa, Medium: 101.3 kPa, High: 116 kPa), which were sustained for approximately 0.67 s. Stimulus magnitude was controlled by changing the pressure inside the balloon, and thus the force applied to the finger. We controlled pressure levels and timing through custom-built Python-based software. This software communicated with a data acquisition (DAQ) system (Quanser Q8-USB; Markham, Ontario, Canada) to monitor and control pressure (KalantaryArdebily et al., 2024). For simplicity throughout the text, we refer to the three stimulus magnitudes as “High”, “Medium”, and “Low” force levels. The force stimuli were jittered with interstimulus intervals ranging from 8 to 12 s (mean: 10 s). Stimulus timing was determined using OptimizeX (Spunt, 2016), with each force level as its own condition.

### 2.3 Functional magnetic resonance imaging

**Fig. 2.**
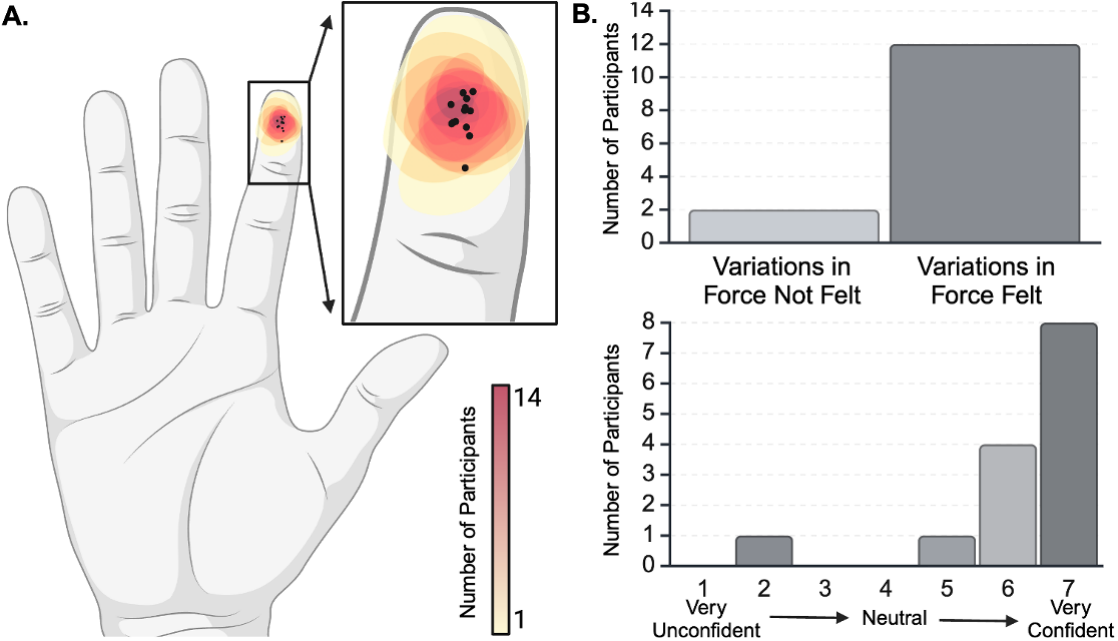
Perception of tactile stimulus. (A) Heat map of participant-reported stimulus location. Participants were instructed to draw a circle indicating the location and relative size of the tactile stimuli. The black dots represent the centroid of each subject’s reported stimulus presentation area. (B) The majority of participants (12/14) perceived variations in stimulus magnitude and were very confident in their answer.

Participants were asked to rest on the MRI table in a head-first supine position with their arms to their sides and their palms lying on their hips (Fig. 1A).^1^ During the scans, participants were instructed to lie still and focus on a visual fixation symbol. They were asked not to cross their arms to avoid tactile perceptual confounds associated with body position (Azañón et al., 2015). We placed the tactile stimulator on the participant’s index finger, centered to match the midline of the distal phalanx. We attached the stimulator using medical tape (Fig. 1B). Data were collected using a 3T Siemens Prisma with a 32-channel head coil. fMRI scans used a multi-echo EPI pulse sequence provided by the Center for Magnetic Resonance Research (CMRR, Minnesota) ($\text{FA}=70^{\circ }$, repetition time (TR) = 2000 ms, voxel size = 1.7 x 1.7 x 4.0 ${\text{mm}}^{3}$, field of view (FoV) = 180 mm, multi-band factor = 2, TE1 = 13.40, TE2 = 39.52, TE3 = 65.62 ms) (Moeller et al., 2010; Setsompop et al., 2012). We collected 34 axial slices. The T1 anatomical used the Magnetization-Prepared Rapid Gradient Echo (MPRAGE) pulse sequence ($\text{FA}=9^{\circ }$, TR = 2300 ms, slice thickness = 1 mm, FoV = 256 mm, multi-band factor = 1, TE = 2.9 ms). All participants received a total of 90 stimuli. The tactile stimuli were applied across four runs with a variable number of stimuli in each run (ranging from 18 to 30 stimuli per run). A total of 552 images were collected across the four runs (run 1:103, run 2:138, run 3:140, run 4:171). This was done to prevent participants from learning the number of events, since they were asked to keep a mental count of the force stimuli detected during each scan to maintain focus on the task, as done in Hämäläinen et al. (2000). The post-scan questionnaire included Likert scales on body comfort, stimulator comfort, and placement, and whether variations in stimulus magnitude were perceived (Fig. 2).

### 2.4 Data analysis

We used AFNI, FSL, and tedana (version 0.0.11; DuPre et al., 2021) to analyze the fMRI data. Preprocessing of the multi-echo data included slice-time correction (3dTshift), rigid-body motion correction to the first time point of the first echo (3dvolreg and 3dAllineate), masking (BET with erosion), and multi-echo combination and de-noising (tedana, see next paragraph). The combined echoes were treated as single-echo data and processed using afni_proc.py. The afni_proc.py pipeline included EPI alignment to the skull-stripped anatomical, normalization to the Montreal Neurological Institute (MNI) template, spatial smoothing with a $4{\text{ mm}}^{2}$ FWHM Gaussian kernel, and voxel-wise signal scaling to a mean of zero.

We used tedana for T2*-weighted echo combination and independent component analysis (ICA) denoising (DuPre et al., 2021). The data were then dimensionally reduced using echo-time-dependent principal component analysis (PCA) (Y.-O. Li et al., 2007). We manually classified independent components from the ME-ICA based on their likelihood of representing BOLD-related activity. Our multi-echo ICA classification pipeline closely paralleled that of Reddy et al. (2024). Our manual classification was based on echo-time dependence, spatial distribution, frequency distribution, and temporal noise (Reddy et al., 2024). Subsequently, the non-BOLD ME-ICA components were recombined as a single covariate to denoise subsequent general linear model (GLM) analyses. Specifically, the sub-matrix of all non-BOLD components was decomposed using PCA and the first eigen-time series was used as a GLM co-variate. The first eigen-time series accounted for greater than 80% of the total variance for all runs and all participants.

We then performed a GLM analysis that consisted of the stimulus timing, six motion regressors, and the non-BOLD eigen-time series co-variate. The three stimulus force levels were included as different task conditions, and stimulus onset and duration were convolved with AFNI’s dmBLOCK HRF. We refer to AFNI’s dmBLOCK as the “standard HRF” as it was designed to characterize a typical positive BOLD response.

### 2.5 ROI analysis

Our primary analysis was based on scrutinizing ROIs within S1, which can be subdivided into cytoarchitecturally and functionally distinct Brodmann’s areas 1, 2, 3a, and 3b (Kaas, 1983). We compared responses between contralateral and ipsilateral BA1, BA2, and BA3b since these areas have been attributed to tactile perception (Rosenthal, 2023), whereas BA3a is thought to be related to proprioception and nocioception (Lutz & Bensmaia, 2021; Whitsel et al., 2019). We defined Brodmann areas 1 and 2 using the Brodmann_MM3dRm atlas, while BA3b was defined using CA_ML_18_MNI (Geyer et al., 2000). To focus on areas of S1 involved in hand/finger representation, analyses of both beta coefficients and time series data were restricted to voxels identified as stimulus responsive at the group level. Specifically, we used the intersection between atlas definitions and whole-brain group-level GLM analysis. We applied a group-level whole-brain mask to include only voxels significantly responsive (FDR-corrected p < 0.05) to the tactile stimuli within each atlas-defined region. These responsive clusters were then segmented according to Brodmann’s area labels. We also examined the percent signal change time course to better understand the temporal characteristics of each ROI. For each participant, we computed the median percent signal change across stimulus repetitions to reduce the influence of outliers. We also baseline corrected each participants’ time series by subtracting the average signal at time points -2 and 0 s from all subsequent time points. We then averaged across participants to obtain group-level values. To parallel this analysis, we also modeled the response pattern using a finite impulse response basis set. We used eight TENT functions with a 0 s start and a duration of 14 s. Each TENT function was separated by TR = 2 s. Using the group-level map, we then performed clustering using AFNI’s 3dkmeans. We included all voxels within the contralateral and ipsilateral S1 to define three primary clusters. This was done for the full atlas-defined region to explore distinct response profiles that may be missed by a standard HRF analysis.

## 3 Results

### 3.1 Perception of tactile stimulus

All participants reported perceiving the tactile stimulus on the distal phalanx of their right index finger (Fig. 2A). The participants’ stimulus counts differed only marginally from the true number of stimuli (0.21 ± 1.05). Although the “high”, “medium”, and “low” force levels were intermixed within each run, the stimulus count errors were significantly less than 1/3 (the number of low force level stimuli), suggesting that all three stimulus force levels were above the perceptual threshold. We found no significant difference between the participants’ reported stimulus counts and the actual number of stimuli applied across the runs (Kruskal–Wallis statistic = 4.156, p = 0.527), indicating that task attention was consistent throughout the runs (Supplementary Fig. S1). After the session, the majority of participants (12/14) reported feeling variations in stimulus force (Fig. 2B).

### 3.2 Distinct responses in contralateral vs. ipsilateral S1

**Fig. 3.**
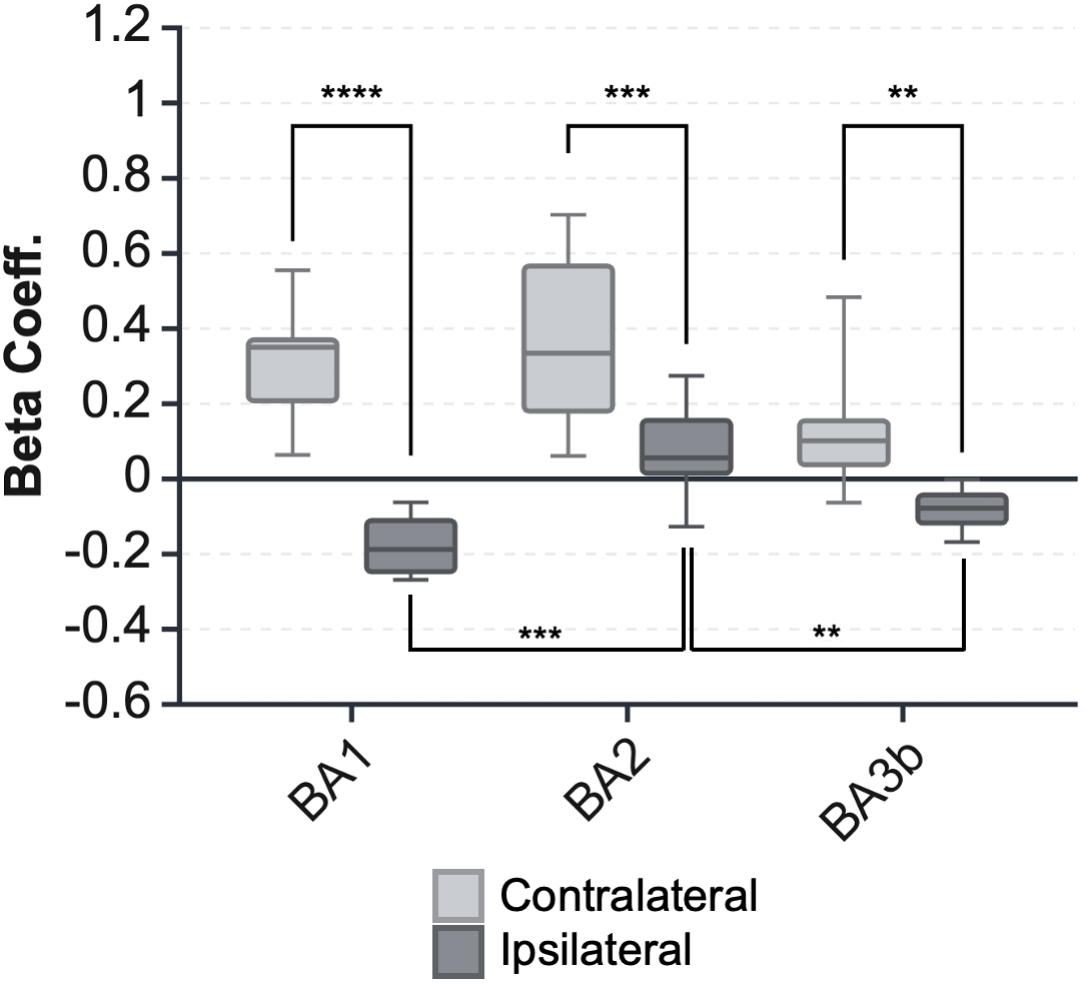
BOLD response magnitude in ipsilateral and contralateral S1. The BOLD response magnitudes were calculated for each participant within each ROI. Stimulus force level was used as a parametric modulator, and the resulting beta coefficient represents the mean signal across the three force levels. Error bars represent the 95% confidence interval across all participants. Asterisks indicate levels of statistical significance (*p < 0.05, **p < 0.01, ***p < 0.001).

As hypothesized, contralateral S1 had a significant positive response while ipsilateral S1 demonstrated a significant negative response. A repeated measures two-way ANOVA with a Tukey correction showed a significant difference between the BOLD response magnitude in the contralateral and ipsilateral hemispheres (F(1, 13) = 81.266, p < 0.0001; Fig. 3). Ipsilateral S1 had a smaller response magnitude across all Brodmann’s areas than the contralateral hemisphere. However, effect size may be inflated by our ROI definition, which used the intersection of atlas-defined ROIs with whole-brain GLM group results (Kriegeskorte et al., 2009). An alternative analysis that avoids this issue, but is less specific to tactile S1, is shown in Supplementary Figure S2 and in the FIR cluster analysis in Fig. 7C, which only used the atlas definitions of these regions.

### 3.3 Brodmann area-specific responses in ipsilateral S1

**Fig. 4.**
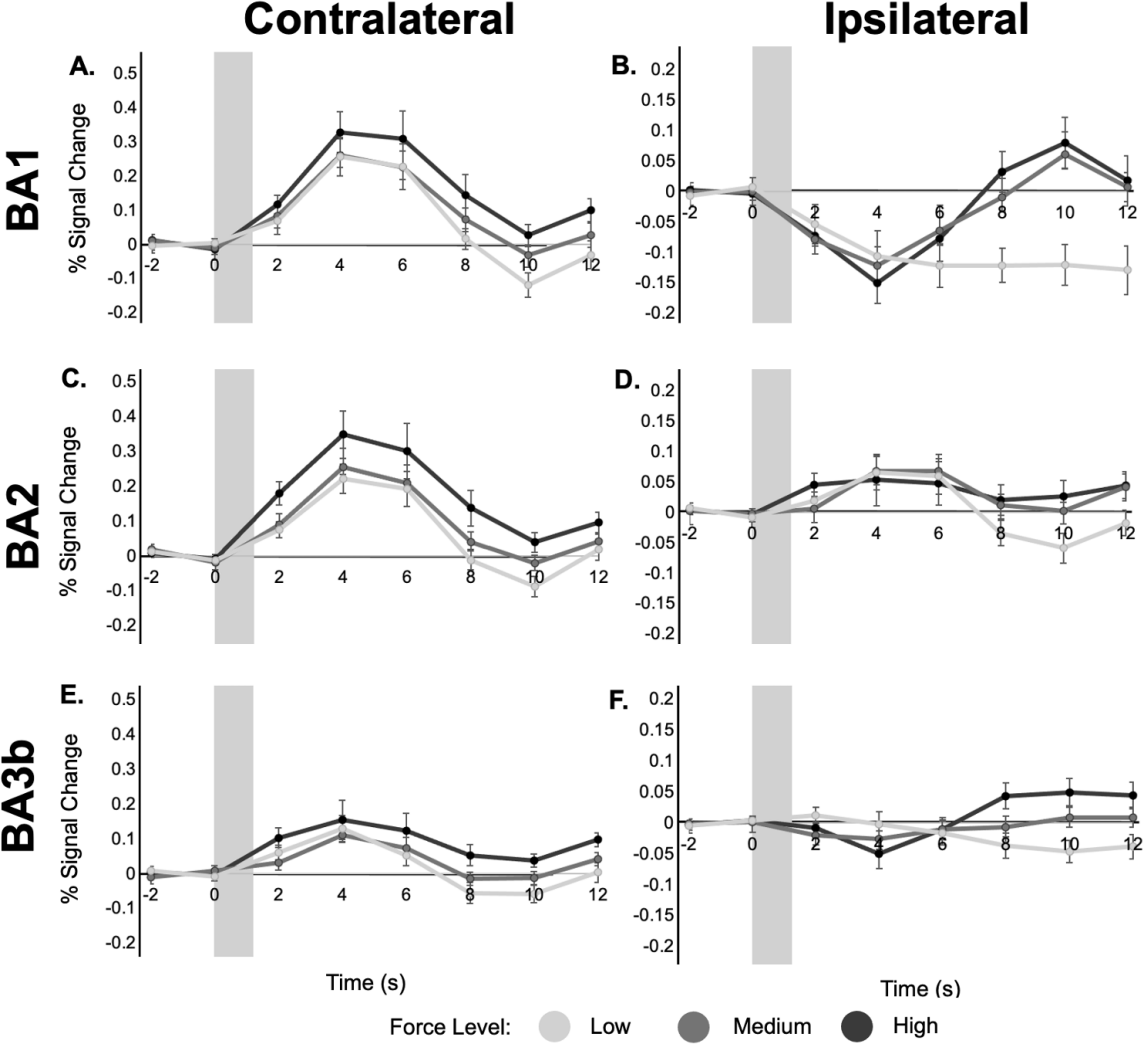
BOLD response time series in ipsilateral (B,D,F) and contralateral (A,C,E) S1. Each point represents the mean signal change within each ROI, and error bars represent the standard error of the mean. Note the vertical axis scale is fixed in each column, but differs between the contralateral and ipsilateral ROI plots. The gray shaded bar represents the timing and duration of the stimulus.

We further hypothesized that within ipsilateral S1, BA1 and BA3b would be the specific subregions with a negative response (Eickhoff et al., 2008; Hlushchuk & Hari, 2006; Klingner, Ebenau, et al., 2011; Klingner et al., 2010, 2014; Schäfer et al., 2012). Testing this, ipsilateral BA1 and BA3b had significantly different response magnitude than ipsilateral BA2 (non-independent statistics, p < 0.0001, p = 0.004591 respectively; Kriegeskorte et al., 2009), indicating a non-homogeneous response pattern within the ipsilateral S1 (Fig. 3). Ipsilateral BA2 had a positive response, similar to contralateral S1 regions, with a positive peak around 5 s after stimulus onset (Fig. 4). In contrast, ipsilateral BA1, and to a lesser extent ipsilateral BA3b, had a *negative* peak around 4 s after stimulus onset. We also observed a potential secondary peak around 10 s after stimulus onset (Fig. 4B, F).

### 3.4 Ipsilateral BA1 reflects biphasic BOLD activity

Based on Figures 3 and 4, we further examined ipsilateral BA1. While Figure 3 showed a robust negative beta coefficient, Figure 4B further suggested that this negative component is followed by a positive overshoot. To assess whether this biphasic response is BOLD related, we compared percent signal change across echo times, and we calculated the T2* time series as follows:

$$S\left(TE_{n}\right)=S_{0}e^{-TE_{n}/T_{2}^{*}}.$$

**Fig. 5.**
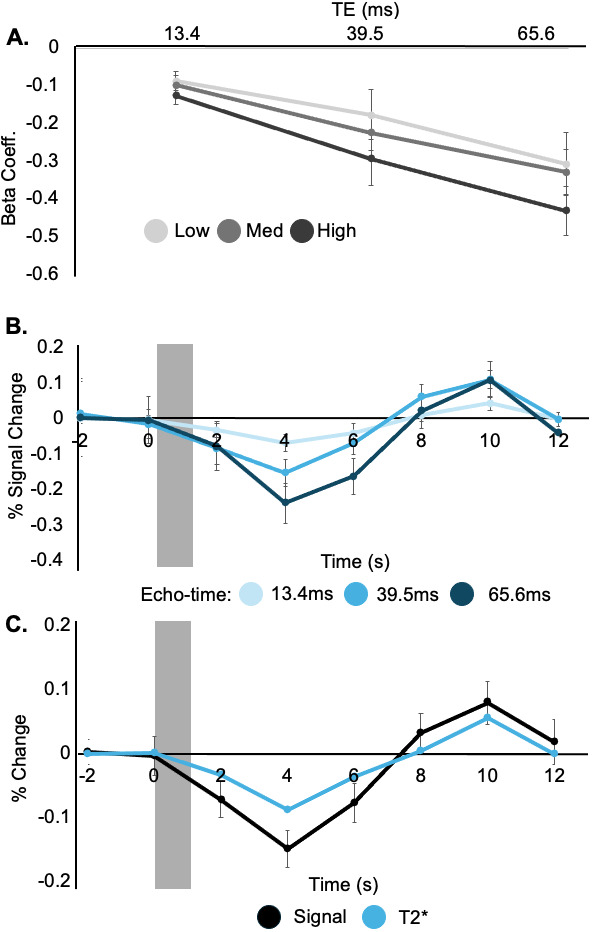
Echo-time dependence in ipsilateral BA1. (A) BOLD response magnitude is larger (more negative) at longer TEs. These beta values were found using the standard dmBLOCK HRF and, therefore, capture the initial negative signal change observed in ipsilateral BA1. (B) Amplitude was monotonically dependent on echo time, with longer echoes having a greater absolute magnitude change. This result indicates that the initial negative response does have echo-time dependence. (C) Both the T2* and percent signal change time series show an initial negative BOLD response followed by a delayed positive response. The similarity of these two time series suggests that both phases are BOLD related. Each point represents the mean, and error bars represent the standard error of the mean across participants. B and C include data from only the high force level, which had the largest initial negative and delayed positive responses.

We only examined the high-force level epochs, based on their robust and consistent negative response. Baseline T2* estimates varied across participants, ranging from 46.6 ms to 59.9 ms. Nevertheless, to account for this variability, both percent signal change and T2* data are presented as percent change from baseline (Fig. 5). In ipsilateral BA1, the response magnitude of both the initial negative phase and the secondary positive phase scaled with TE (Fig. 5A). Longer TEs had larger response magnitudes (Fig. 5B). Additionally, we observed a similar biphasic response pattern between the signal change and the T2* time series (Fig. 5C). Note that while we focused on the largest force level epochs, we repeated this process for all force levels. For the low force level, we observed that the initial negative response exhibited TE dependence similar to the other force levels. In contrast, the sustained negative signal change plateau observed in response to the low force level stimulus (Fig. 4) was not TE dependent, and a similar plateau was not seen in the T2* response time series, indicating that this is likely not a BOLD signal effect. However, we do not have an alternative explanation for this potential artifact.

### 3.5 Modeling biphasic BOLD in ipsilateral BA1

**Fig. 6.**
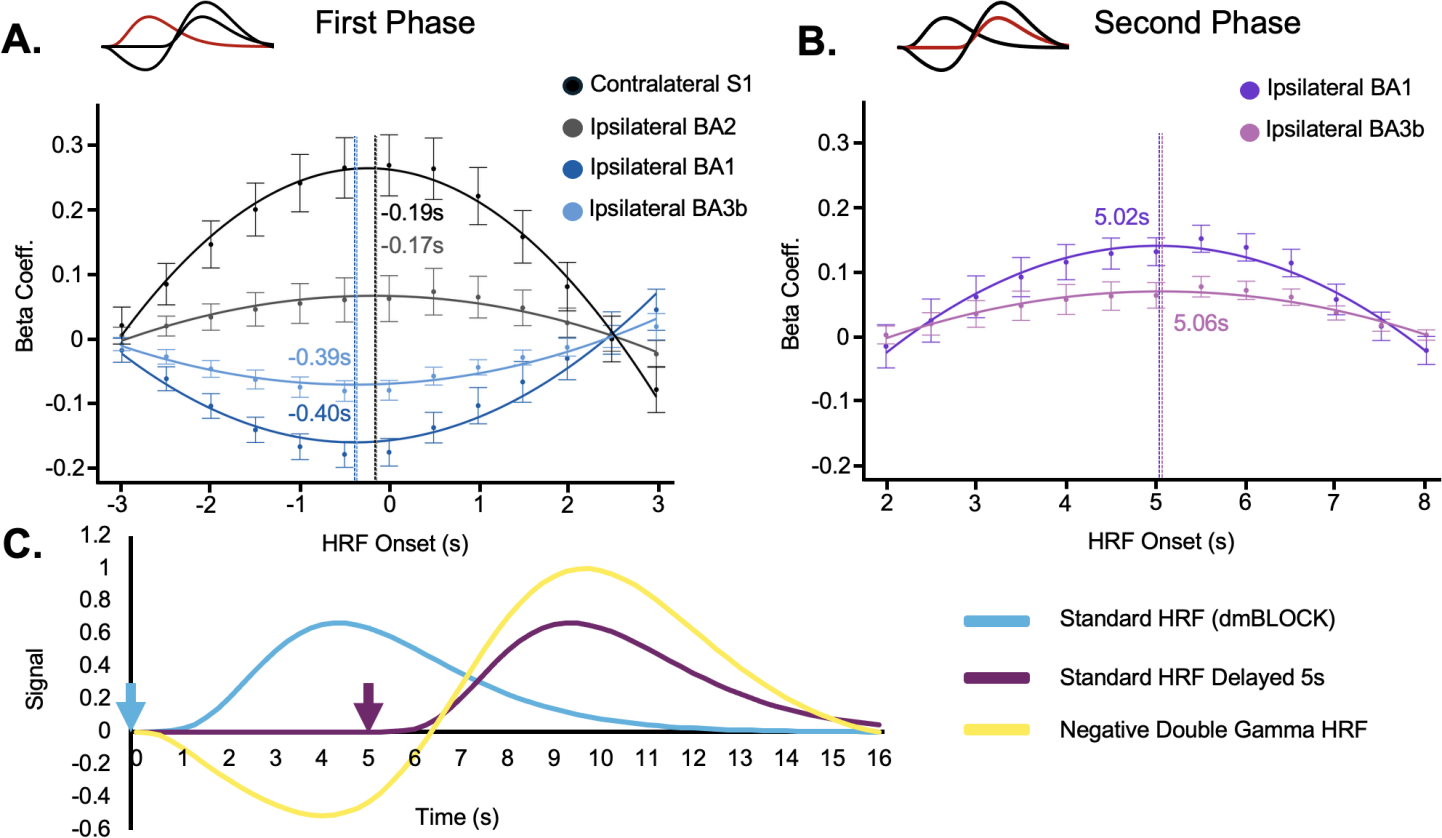
Characterizing the biphasic response. (A, B) Examine the delay in the initial and secondary BOLD response phases, respectively. To do this, we performed GLM analyses (collapsing across all force levels) with a standard HRF (AFNI’s 3dDeconvolve’s dmBLOCK option) across a range of HRF onset times. (A, B) The GLM fit magnitudes as a function of stimulus timing delay. The resulting beta coefficient versus delay plots shows the mean and standard error of the mean across participants. A higher beta coefficient indicates a better fit between the HRF model and the observed BOLD response. A second-order polynomial was fit to the beta coefficients to estimate the magnitude and delay of the BOLD response (see corresponding polynomial curves). The resulting delay for each ROI is marked with a vertical dotted line. (A) Contralateral S1 and ipsilateral BA2 produced positive beta values, while ipsilateral BA1 and BA3b produced negative beta values. Because we fit these data, we were able to estimate their relative delays (as annotated); however, all the delays were approximately 0 s, especially in the context of a 2 s TR. (B) We then characterized the secondary response phase observed in ipsilateral BA1 and BA3b. As shown, the optimal HRF onset was approximately 5 s after the initial stimulus. In other words, if we were to model only the secondary positive response phase in ipsilateral BA1 and BA3b, we would use stimulus times delayed by approximately 5 s. (C) Based on the timing above, a 0 s delay produces the light blue curve and the 5 s delay produces the purple curve using dmBLOCK. To approximate both these timings, and the negative magnitude of the initial phase in ipsilateral BA1 and BA3b, we tested a negative double gamma HRF (yellow curve). Arrows indicate the HRF onset time for each model.

To estimate the two response phases, we estimated the optimal delay for each peak, following methods similar to Liao et al. (2002). We conducted a delayed HRF analysis to identify the optimal HRF delay that best matched the time series data from these regions. We tested various delay intervals, adjusting the standard HRF onset by 0.5 s steps within a 3 s range around the apparent peak BOLD response, as shown in the time series plots (Fig. 4B). The 6 s window was chosen to capture a broad range of timings without confounding effects from the negative portion of the HRF curve. For each delay, a regression model was applied with stimulus force as a parametric regressor. The resulting beta coefficients indicated the quality of the model fit, with higher values suggesting a better fit. A second-order polynomial curve was then fit to the group-level data, and the “ideal” delay was defined as its peak (Fig. 6).

In contralateral S1, Klingner, Ebenau, et al. (2011) suggested that negative BOLD may have a different time-to-peak. Thus, they use a standard HFR delayed by 1 s to approximate this negative BOLD response. To quantitatively assess any differences in response timing between positive and negative BOLD, we included regions with known positive BOLD responses in this analysis. In Figure 6A, we found that in ipsilateral BA1, the negative peak occurred approximately 0.4 s earlier than the standard HRF would predict. In ipsilateral BA1, the maximal beta coefficient corresponded to a delay of approximately 5 s from stimulus onset (Fig. 6B). Based on this 5 s delay, we examined a biphasic HRF using a double gamma approximation with an initial negative phase and a secondary positive phase (Fig. 6C). The 3dDeconvolve negative double GAMMA parameters we used were TWOGAMpw (9.06, 6.06, 0.41, 5.22, 10.31), where the first gamma’s peak and width were 9.06 and 6.06, respectively. The secondary gamma’s peak and width were 5.22 and 10.31, respectively, and the second gamma was scaled by a factor of 0.41.

**Fig. 7.**
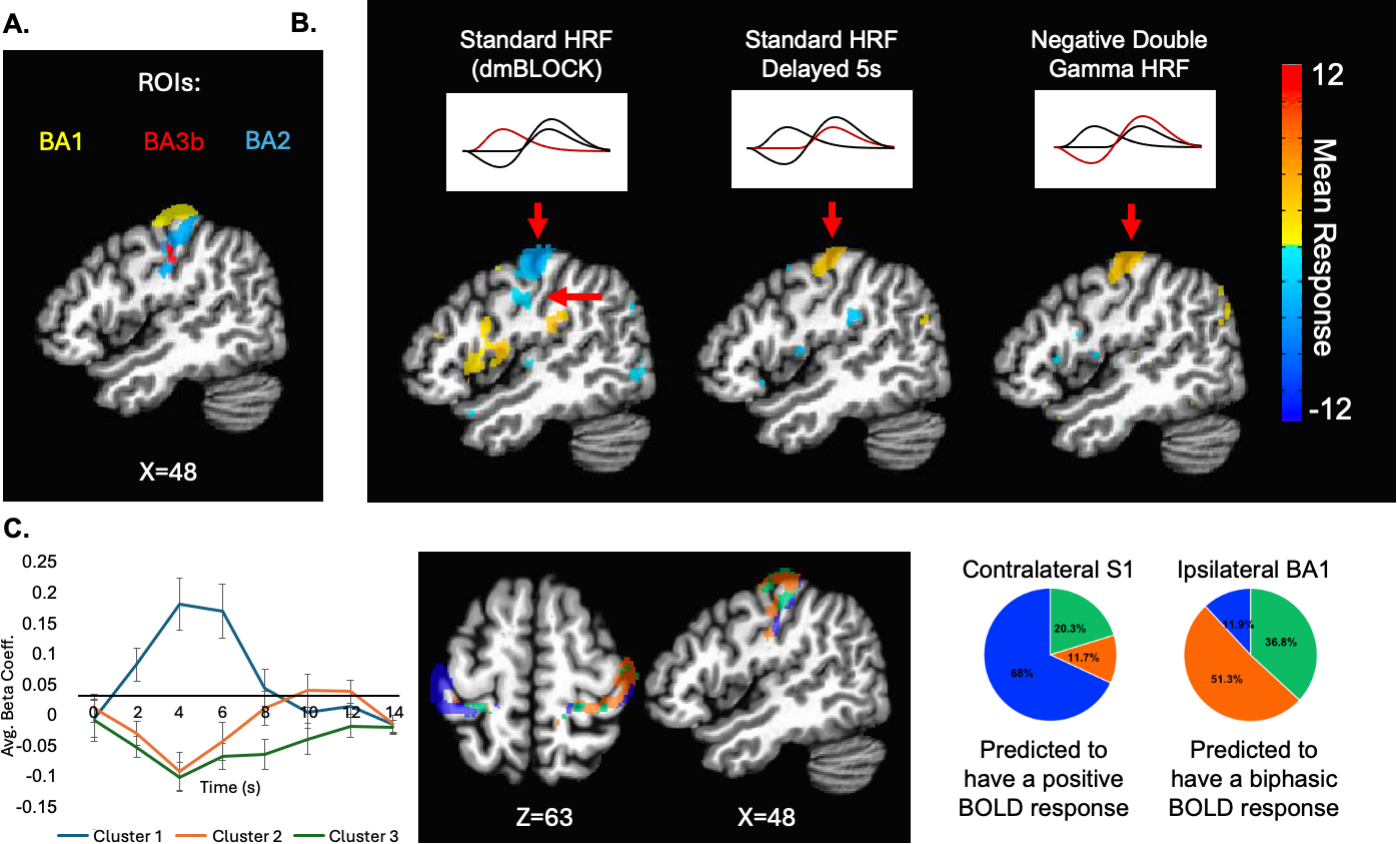
Improved ipsilateral BA1 specificity with the biphasic model. (A) Ipsilateral BA1 and BA2 (defined by the Brodmann_MM3dRm atlas) and BA3b (defined by the CA_ML_18_MNI). (B) Maps generated from the standard HRF (dmBLOCK), the delayed HRF, and our proposed biphasic negative double gamma HRF from Figure 6. Arrows indicate GLM results in ipsilateral S1. Note that the sign of the response changes between the standard HRF (dmBLOCK) and the negative double gamma HRF reflecting their negative and positive correlation with the BOLD time series, respectively. Maps were thresholded at a voxel-wise FDR-corrected p < 0.05. (C) Our finite impulse response analysis defined three distinct response patterns. Paralleling our previous findings, we see a robust biphasic response. The mean response for each of these clusters is plotted with error bars representing the standard error of the mean. We can also examine how these response patterns are spatially organized within the anatomical ROIs. We see that the response pattern that resembles a positive BOLD response (cluster 1) is most prevalent in contralateral S1, as would be expected. The biphasic response cluster (cluster 2) is the most prevalent response pattern in ipsilateral BA1. See also Supplementary Figure S3.

We then compared a series of GLM maps to assess how each of these HRFs captured the distinct response pattern we observed in ipsilateral S1 (Fig. 7). With the standard (dmBLOCK) model, we observed a large negative cluster near ipsilateral (right) BA1. In contrast, when using the delayed HRF to examine the secondary positive response phase, we observed a smaller positive cluster in ipsilateral S1. With the negative double gamma HRF approximation, we observed a positive cluster in a similar location. This cluster analysis helps identify the spatial specificity of the biphasic response and corroborates the notion that the secondary positive phase is specific to ipsilateral S1 (Fig. 7B). We see a reduced response area when using the negative double gamma HRF that better resembles the expected location and shape of BA1, suggesting that the negative double gamma HRF exhibits more specificity than the standard HRF (dmBLOCK).

To ensure that we captured significant signal changes that were time locked to the stimulus, we complemented the biphasic modeling shown in Figure 7B, by performing a Finite Impulse Response (FIR) analysis. Unlike the standard HRF approach, the FIR model does not assume a fixed temporal structure. We performed this analysis within the full Brodmann’s atlas defined contralateral and ipsilateral S1 to avoid bias associated with our ROI definitions. We found three distinct response patterns within S1 (Fig. 7C; Supplementary Fig. S3). The first cluster has a response time series that resembles a typical positive BOLD HRF with a positive peak 4–6 s after stimulus onset. This response pattern is most prevalent in the contralateral S1 and ipsilateral BA2. The second cluster parallels our biphasic HRF findings, with a negative epoch at 4 s and a delayed positive peak 10–12 s after stimulus onset. The final cluster resembles a negative BOLD response with a negative epoch at 4 s. The biphasic and negative response patterns are more prevalent in ipsilateral BA1 and BA3b. The negative double gamma HRF is highly correlated with the biphasic response pattern found in the second cluster of our FIR analysis (r(6)=0.8799, p<0.00001 ).

### 3.6 Force-dependent temporal dynamics

**Fig. 8.**
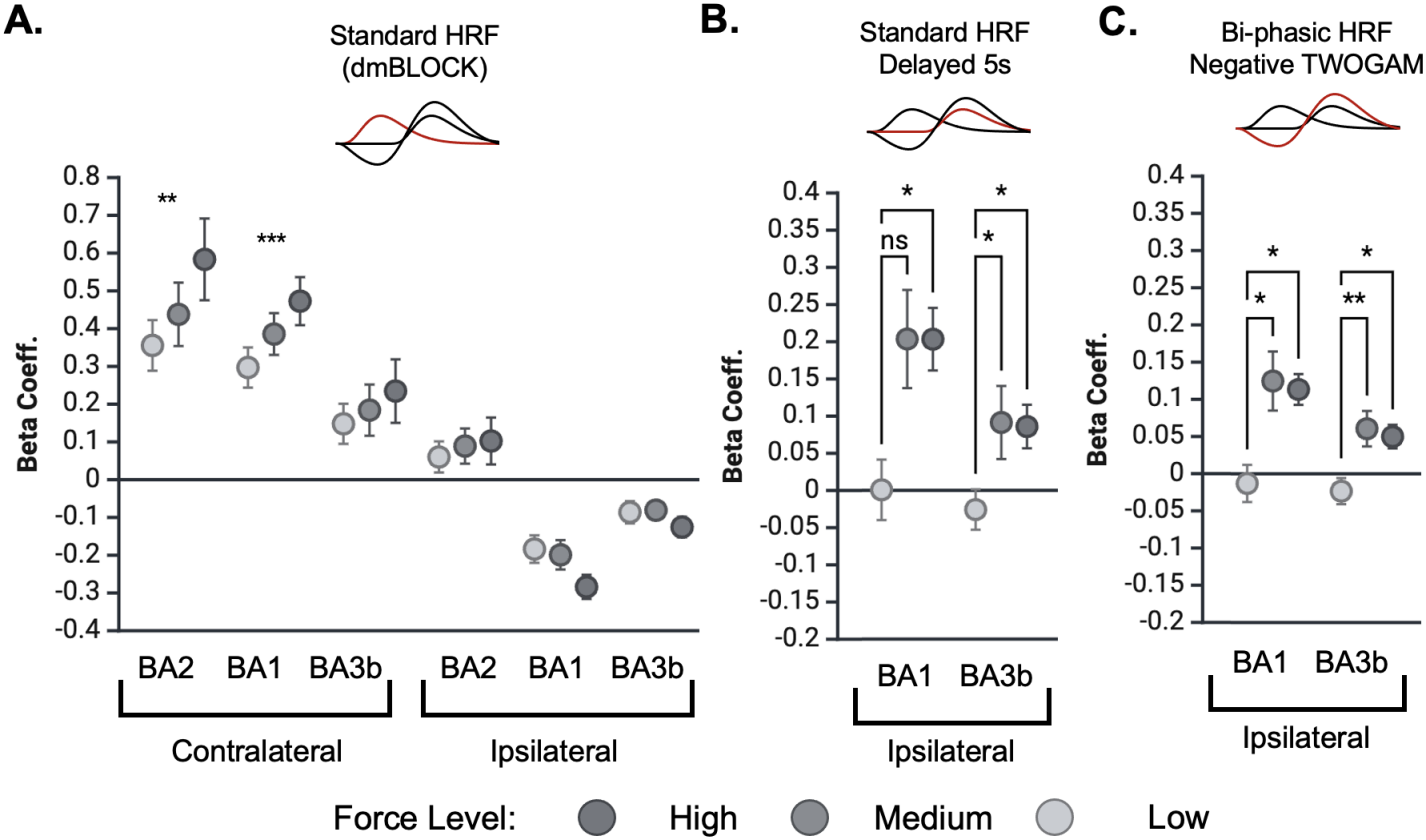
Stimulus force modulates positive contralateral and biphasic ipsilateral BOLD responses. Shown are the responses for high, medium, and low force levels in S1. Each point represents the mean response magnitude across subjects, and error bars denote the standard error of the mean. (A) The standard (dmBLOCK) HRF responses. In contralateral S1 we see a positive relationship between stimulus force level and response magnitude. In contrast, none of the ipsilateral regions demonstrated force level dependencies. (one-way ANOVA; **p < 0.01, ***p < 0.001). (B) The secondary response phase differs with stimulus force level. (C) The negative double gamma HRF responses in the ipsilateral S1 regions with negative BOLD. While not as sensitive as contralateral responses, the negative double gamma fits show enhanced sensitivity to force level compared with the ipsilateral fits in A (one-way ANOVA with pairwise comparisons using Tukey’s correction; *p < 0.05).

We compared both the standard HRF and negative double gamma HRF to evaluate our ability to detect stimulus force. We performed hemisphere and HRF-specific two-way ANOVAs of region (BA1, BA2, BA3b) and force level (low, medium, high). For the standard HRF, we examined both hemispheres. In contralateral S1 (BA1, BA2, BA3b), we observed a significant main effect of stimulus force level (F(2, 26) = 6.476, p = 0.0052). Pairwise comparisons with Tukey’s multiple comparison correction show a significant difference between the low and high force levels in the contralateral BA1 (p = 0.0173) and the medium and high force levels in BA2 (p = 0.03546). In contrast, however, the standard HRF did not produce a significant effect of stimulus force in ipsilateral S1 (F(2, 26) = 0.685, p = 0.5127; Fig. 8A). For the negative double gamma HRF, we focused on this ipsilateral BA1 and BA3b (where the “negative BOLD response” is relevant). In this case, we did observe a significant effect of stimulus force (F(2, 26) = 7.425, p = 0.0028; Fig. 8B). This difference was present even with the inclusion of BA2, which did not exhibit a negative BOLD response in this experiment (Fig. 4D).

**Fig. 9.**
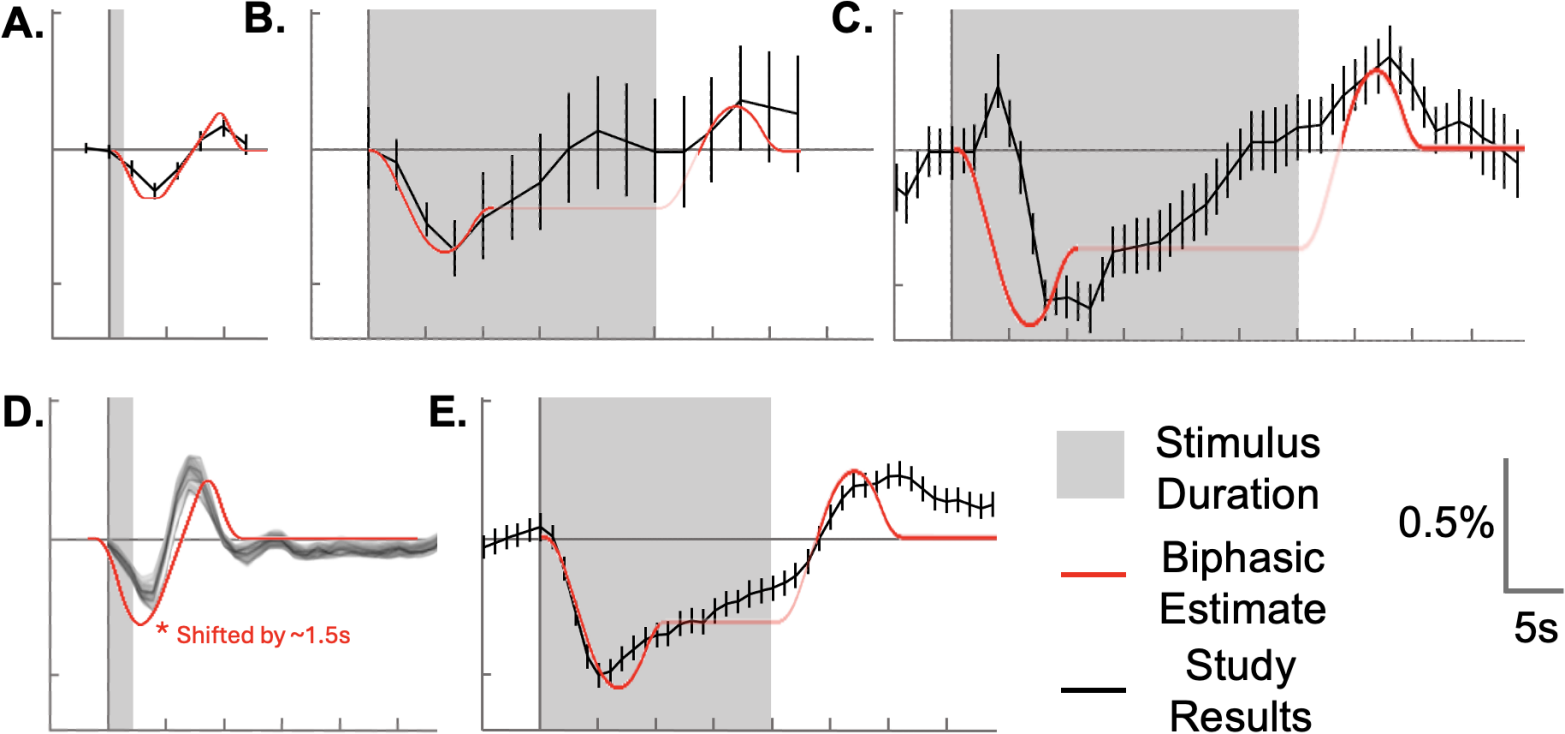
Qualitative comparison across tactile and visual studies. A piecewise linear biphasic HRF was convolved with the stimulus duration of prior studies (red curves in A–E). We qualitatively compared this with fMRI signal changes from several studies (black curves in A–E). When the biphasic HRF is convolved with long stimulus blocks, it can produce a plateau artifact between the two peaks. To emphasize the timing of the peaks, we made the plateau segment fainter in B, C, and E. The studies include tactile stimulation from (A) the current study (Fig. 5C), (B) Hlushchuk and Hari (2006, modified from Fig. 2), and (C) Kastrup et al. (2008, modified from Fig. 3b). In addition, we show responses to visual stimulation from (D) de la Rosa et al. (2021, modified from Fig. 2), and (E) Shmuel et al. (2006, modified from Fig. 1d). In each of these studies, the response line (black) was created by tracing over plots from each published work. For the de la Rosa study, our HRF was shifted 1.5 s earlier to align with the reported data. (Black curves adapted from the corresponding studies).

As a final test of our hypothesis, we compared a biphasic negative BOLD HRF with the stimulus duration found in previously published tactile and visual studies (de la Rosa et al., 2021; Hlushchuk & Hari, 2006; Kastrup et al., 2008; Shmuel et al., 2006; Fig. 9). As mentioned, most prior reports have used block designs. While the TWOGAMpw parameters are easy to evoke in AFNI and were appropriate for our event-related dataset, this parameterized negative double gamma model produces an exaggerated positive plateau between the initial negative and subsequent positive peaks when convolved with longer stimulus durations. Thus, to compare with those past reports, we did not use the general purpose TWOGAMpw approximation, but instead used a fixed-TR, piecewise linear model to produce Figure 9. The temporal profile of our negative double gamma and piecewise linear HRFs is highly correlated (r(56)=0.9313, p<0.00001 ), but handles the transition between the initial negative and delayed positive phases differently. Specifically we used a sampling rate of 0.25 s and an HRF with the following parameters in MATLAB ([[0 0] [-.2:-.2:-1.6] [-1.6* ones(1,7)] [-1.5:0.15:1.4] [1.3:-0.2:0.1]]) to conduct a qualitative comparison of those past studies (for comparison with Fig. 9, see Supplementary Fig. S4).

Figure 9A shows our average response to the “high” force-level stimulus in ipsilateral BA1 from Figure 4B, along with the piecewise linear HRF convolved with our stimulus duration. In Figure 9B–E, we similarly convolved the piecewise linear HRF with the stimulus duration specific to each study. For these block designs, we convolved with a simple square wave; thus, the convolution result includes a plateau proportional to the length of the stimulus block. We made this plateau transparent in Figure 9B–E. Figure 9B shows a negative BOLD response elicited in ipsilateral S1 using a 25 s tactile stimulus (Hlushchuk & Hari, 2006). Similarly, Figure 9C shows an example from ipsilateral S1 from a 30 s block-design electrotactile stimulus to the right median nerve (Kastrup et al., 2008). In visual cortex, Figure 9D shows results from a short duration (2 s) visual stimulus eliciting responses in ipsilateral primary visual cortex (V1) (de la Rosa et al., 2021) (note, in this case, we shifted our piecewise linear convolution result by 1.5 s). This 1.5 s dependency may have come from a few sources, including our misinterpretation of de la Rosa et al. (2021) stimulus timing. Finally in Figure 9E we show a 20 s block-design response in peripheral V1 (Shmuel et al., 2006). Some general observations from Figure 9 include the fact that the initial negative phase of the piecewise linear biphasic HRF aligns well with the time series observed in Hlushchuk and Hari (2006), de la Rosa et al. (2021), and Shmuel et al. (2006). Despite the 1.5 s dependency, the de la Rosa et al. (2021) results nevertheless demonstrate strong similarity between the two waveforms. Notably, our biphasic HRF predicts a delayed positive response in each of these studies that, while present, was only noted in the de la Rosa et al. (2021) article. While qualitative in nature, Figure 9’s cross-study and cross-modal comparisons add further evidence supporting the plausibility of this study’s biphasic hypothesis.

## 4 Discussion

### 4.1 Discussion of results

In response to unilateral tactile stimuli, we observed bilateral responses in primary somatosensory cortex (S1; BA1, BA2, BA3b). As hypothesized, ipsilateral S1 exhibited distinct BOLD response patterns. Consistent with prior findings (Eickhoff et al., 2008; Hlushchuk & Hari, 2006; Klingner, Ebenau, et al., 2011; Klingner et al., 2010, 2014; Schäfer et al., 2012), we observed a positive BOLD response in ipsilateral BA2, while ipsilateral BA1 and BA3b exhibited negative BOLD responses.

Extending previous studies, our results and review of past reports led us to hypothesize that the ipsilateral responses are not simply “negative”, as has been previously characterized. As shown in Figure 4B, the signal in ipsilateral BA1 was not well characterized by a single negative deflection. Instead, we propose that this response is biphasic in nature, with an initial and secondary period of neural activity. To assess whether the biphasic response was truly BOLD related, and could, therefore, be driven by neural activity, we used multi-echo fMRI, examining signal changes across three echo times. This method has been used previously to differentiate BOLD from non-BOLD signal components (Devi et al., 2022; Havlicek et al., 2017; Kundu et al., 2012; N. Li & Jasanoff, 2020). Notably though, this approach has been used primarily in the context of positive BOLD responses. There remains limited exploration of echo-time dependence in negative BOLD responses, a gap our work begins to address. In ipsilateral BA1, both the negative and positive phases of the signal showed echo-time dependence (Fig. 5B). We also observed a biphasic T2* response that paralleled the mean signal, reinforcing the interpretation that both components of the biphasic response are BOLD related and reflect changes in local blood oxygenation (Peltier & Noll, 2002; Serai, 2022).

To model this biphasic response, we used two biphasic HRF models: a negative double gamma function and a piecewise linear function. While time-to-peak and undershoot amplitude differ slightly in these two HRF models, they both function similarly in approximating our biphasic response. More precise future HRF models will require more data with better temporal precision. It would also be beneficial to fit such models with multi-modal stimuli that sample various stimulus durations. Compared with a standard HRF model, the negative double gamma HRF improved stimulus response specificity within ipsilateral BA1 (Fig. 7B). To complement these HRF analyses, we also estimated the stimulus-locked signal for every voxel within the S1 region using AFNI’s TENT functions (eight TENTs per voxel, each separated by 2 s), Figure 7C. These TENT coefficients were then clustered (3dkmeans) to identify the primary response profiles present in S1. This analysis revealed three distinct clusters: a typical positive BOLD response, a sustained negative BOLD response, and a negative-to-positive biphasic response. The emergence of this biphasic pattern through a flexible, unbiased model confirms that it is a robust, stimulus-locked feature of the ipsilateral S1 response and not an artifact of a canonical HRF template. Given that the negative double gamma HRF was derived from our data, we further sought to evaluate whether similar patterns generalize to past studies. To do this, we convolved the biphasic HRFs with the stimulus timings used in prior reports. The results in Figure 9 and Supplementary Figure S4 corroborate a biphasic response was present in both tactile and visual studies. This distinct biphasic response pattern also has been observed and discussed by de la Rosa et al. (2021), where they described an initial negative response followed by a “strong but significantly later positive peak.” Their study used both block design and event-related visual stimuli to examine the negative BOLD HRF. The present study extends this work by framing negative BOLD explicitly as “biphasic,” suggesting parameters to model the HRF and using multi-echo data to confirm that both phases of the response are BOLD related (Fig. 5).

Our results indicate that the BOLD response to varying stimulus force levels exhibits both spatial and temporal specificity. In contralateral S1, BOLD amplitude was modulated by stimulus force magnitude, as has been previously reported (Backes et al., 2000). In ipsilateral S1, however, we did not observe force effects with a standard analysis. Instead we see no secondary response at the low force level and similar secondary response magnitudes at the “medium” and “high” force levels (Fig. 8). Notably, the response magnitude across force levels did not vary monotonically in the ipsilateral cortex (Figs. 4B and 8B). Instead, we see no response at the low force level and similar response magnitudes at the “medium” and “high” force levels (Fig. 8B). Based on the small range separating “low” vs. “high” force levels, one possible explanation for the similarity between “medium” and “high” is that our current fMRI methods are not sensitive enough to decode differences in these two force levels. However, we do not think that the similarity in response magnitudes reflects a vascular ceiling effect because “low” did not elicit a response above baseline, and physically and perceptually the difference between “low” and “high” force levels is not large. Nevertheless, these results suggest that there could be a minimum stimulus force threshold that needs to be exceeded to elicit a biphasic response in ipsilateral BA1 and BA3b. To fully understand the impact of stimulus force in ipsilateral S1, it is essential to consider the entire biphasic BOLD response, as the initial phase on its own does not differentiate between force levels. It may also be important to examine how stimulus properties influence the biphasic response in different sensory domains. For example, de la Rosa et al. (2021) found that the initial negative BOLD response magnitude in the primary visual cortex varied significantly with visual stimulus features (i.e., eccentricity), at odds with our observation of no force dependence in the analogous response in primary sensory cortex.

Speculating on its functional significance, the early negative phase in BA1 may reflect rapid, stimulus-driven suppression mediated by intrahemispheric inhibitory mechanisms. Given the strong anatomical connectivity between BA2 and BA1, it is possible that initial excitation in BA2 engages feedforward inhibitory circuits that transiently suppress BA1. This suggests a gating or contrast-enhancement function, momentarily suppressing non-relevant or spatially diffuse signals to enhance the fidelity of tactile representations (Schäfer et al., 2012).

While the secondary phase may arise simply from a vascular rebound effect, both its consistent timing and its BOLD dependence suggest that it is neurally driven. We propose that the secondary phase is driven by stimulus cessation. Ipsilateral BA1 is held in a sustained inhibitory state during stimulation, with stimulus offset triggering release from inhibition and a delayed positive BOLD response. Supporting this view, Figure 9 shows that the positive peak occurs 7.5–10 s after stimulus offset, independent of stimulus block length or sensory modality. This offset-locked release from inhibition may gate delayed excitatory input to ipsilateral BA1, potentially arising from polysynaptic intrahemispheric pathways or feedback from higher-order regions such as S2 (Blatow et al., 2007; Chung et al., 2014). The secondary phase appears crucial for distinguishing between different magnitudes of force involved in touch (Fig. 8B). This later phase separates the signals based on strength of the stimulus, a process that is essential for fine-grained discrimination. The observation that this second phase is responsible for dissecting these subtle differences in force strongly supports the hypothesis that it is a key component of a serial processing pathway, where information is analyzed sequentially to extract more complex features of the sensory experience. This biphasic response profile challenges the traditional view of ipsilateral S1 as passive or purely inhibitory. Instead, it could suggest that ipsilateral BA1 participates in a temporally structured process of tactile encoding, where early inhibition may serve to sharpen response selectivity (Kastrup et al., 2008; Schäfer et al., 2012), and late excitation may reflect integration of higher-order or bilateral sensory cues (Hämäläinen et al., 2000; Klingner et al., 2016; Tamè et al., 2015).

Continued observations across multiple measurement modalities and sensory systems are needed to more generally relate neural activity to BOLD responses. In particular, studying multiple sensory systems would aid in improved characterization of the biphasic ipsilateral response, while also providing multiple windows to interpret its functional meaning (Nelson & Mayhew, 2025). Previous explicit observations of negative BOLD’s positive overshoot are rare (as a notable exception, see de la Rosa et al., 2021). Even more rare has been the attempt to seek mechanistic interpretations, but Gouws et al. (2014) demonstrated that a significant portion of the positive overshoot in visual cortex could be explained by post-stimulus eye blinks. Their interpretation was that the positive overshoot was, at least in part, driven by an increase in blinking after stimulus cessation. We hypothesize that currently unobserved analogous behavioral and/or physiological events exist for ipsilateral S1, and for negative BOLD more generally. As an alternative explanation to the Gouws et al. (2014) results, we hypothesize that instead of blinks causing increased overshoot, the intrinsic overshoot component of the biphasic BOLD response could indicate a “release from inhibition” that enables blinking to resume. The increase blink rate could reflect a compensation for the initial suppression of blinks. Although blink rate explained the subsequent positive BOLD phase in their data, further inspection of their results suggest that it also explained the initial negative phase as well. In sum, our findings suggest that the initial negative BOLD response may not have a simple, direct relationship with a single stimulus or physiological event. This complexity underscores the value of adopting a multimodal approach to study these systems. By integrating data from various sources, neural, physiological, and behavioral, we can gain a more complete understanding of brain activity and the intricate mechanisms behind the BOLD signal’s different phases. This approach would benefit the study of sensory systems and negative BOLD responses in general.

#### 4.1.1 Limitations and future direction

One possible confounding factor complicating previous findings of negative BOLD in ipsilateral S1 is the variety of stimulation methods and stimulus features used. Somatosensory perception involves the complex integration of various stimulus properties, including indentation depth, stimulus frequency, and stimulus modality (e.g., brushing, electrotactile, static pressure). Differences in stimulation modality have the possibility of impacting the patterns of ipsilateral S1 activation observed in fMRI studies (Lipton et al., 2006). In this experiment, we used a force-based tactile stimulus with a short stimulus duration. This offered the advantage of stimulating mechanoreceptors, in contrast to electrotactile stimuli, which acts on the nerve itself. Despite this limitation, we find that our tactile biphasic HRF generalizes beyond tactile stimuli and is able to approximate negative BOLD responses in the visual cortex. Additionally, the precise tactile stimuli applied by our device allow better control of stimulus timing and magnitude. Further innovation in device design of this and similar devices could enable us to target specific mechanoreceptor types.

One limitation of our analysis was our approach to defining S1 ROIs. To obtain an index-finger-specific signal, we used the intersection of atlas-defined ROIs with whole-brain GLM group results. Thus, the effect sizes suggested in Figure 3 may be inflated (Kriegeskorte et al., 2009). To assess how our approach impacted the results, we also examined the larger, less specific atlas-defined Brodmann’s areas. This control analysis yielded comparable patterns of response across hemispheres and Brodmann’s areas (Supplementary Fig. S2). We did not find differences in the beta coefficients (F(1, 13) = 2.868, p = 0.1142). However, the overall response magnitudes were attenuated (Supplementary Fig. S2). This attenuation likely reflects the exclusion of voxels not specific to index-finger/median nerve stimulation. Further work could be done to identify anatomical landmarks for the hand-related sensory-motor regions, which could lead to more refined ROI analyses (Muellen & Schweizer, 2025).

While our multi-echo analyses suggested that the biphasic response is BOLD related, this does not eliminate the possibility of vascular confounds. The BOLD signal is sensitive to a range of hemodynamic variables, including blood flow, volume, and hemoglobin oxygenation (Kim & Ogawa, 2012), which may introduce artifacts independent of neural activity (Harel et al., 2002). Thus, although the T2* and signal time courses agree, additional methods (e.g., concurrent cerebral blood flow measures) could help disentangle vascular and neural signal components. While our protocol utilized a 4 mm slice thickness to achieve a higher signal-to-noise ratio through larger voxel sizes, future work should more extensively explore how this and other acquisition parameters affect the sensitivity required to detect subtle negative BOLD signals. To complement this acquisition strategy, we employed multi-echo combination to compensate for spatial inhomogeneity and signal dropout. Additionally, our multi-echo denoising is thought to minimize non-BOLD artifacts. We employed a conservative denoising approach rather than the widely used method implemented in tedana. Tedana by default regresses out all rejected components. In our dataset, however, tedana identified a large number of non-BOLD components. Including each of these separately would have substantially expanded the GLM design matrix. To address this, we used a PCA-based approach to collapse the tedana-identified non-BOLD components into a single nuisance covariate. This strategy is analogous to methods that use the first eigen-time series to account for other nuisance sources, such as motion, in fMRI analyses (Beall, 2010). Our single nuisance covariate accounted for more than 80% of the variance across tedana components for each run and subject. While we did not aim to optimize multi-echo denoising parameters in this study, we recognize this as a rapidly evolving area of neuroimaging. To facilitate the development and testing of more robust denoising strategies, we have made our raw multi-echo data available via open access https://openneuro.org/datasets/ds006758/versions/1.0.0, allowing for future reanalysis as denoising methodologies continue to mature.

Regardless of its origins, the biphasic response seems to be robust, especially for greater stimulus force levels (Fig. 8). At the same time, though, it has been a largely overlooked feature of ipsilateral BA1 (Fig. 9). While our current study used an event-related design, mapping ipsilateral S1 responses has been historically challenging. Thus, the majority of previous studies have relied on block designs, which are known to elicit more robust BOLD responses. A consequence of this, however, is that the convolution of a block design with an HRF obscures the HRF shape more than an event-related design does. But, as Figure 9B shows, our proposed biphasic HRF is qualitatively consistent with previous studies in S1 and visual cortex. Future work could refine the biphasic HRF parameters that we proposed here by comparing across a variety of sensory modalities and stimulus designs.

## 5 Conclusion

This paper focused on the negative BOLD response in ipsilateral S1, with a primary focus on the more robust BA1. In our ROI analysis, our observations led us to hypothesize that BA1’s response is actually biphasic, particularly for the “medium” and “high” stimulus force levels. By demonstrating echo-time dependence and modulation with stimulus force level, we provided further evidence that the biphasic response in ipsilateral BA1 reflects BOLD contrast mechanisms and is likely neural in origin. Our findings extend prior negative BOLD findings that focused largely on the initial negative response. Finally, the biphasic nature and force sensitivity of ipsilateral S1 suggest it has a more dynamic role in tactile processing than previously recognized.

## Supplementary Material

Supplementary Material

## Data Availability

All MRI data and processing scripts are available at: https://openneuro.org/datasets/ds006758/versions/1.0.0.

## Footnotes

1 Figures 1-3 and 8 were made with BioRender.
