# Supplementary material for: Tactile force evokes a biphasic BOLD response in ipsilateral primary somatosensory cortex
Source: Imaging Neurosci (Camb). 2026 May 21;4:IMAG.a.1236. doi: 10.1162/IMAG.a.1236 (PMC13195919; doi:10.1162/IMAG.a.1236)
Supplement: Supplementary Material [file IMAG.a.1236_supp.pdf]

1 Supplemental Material

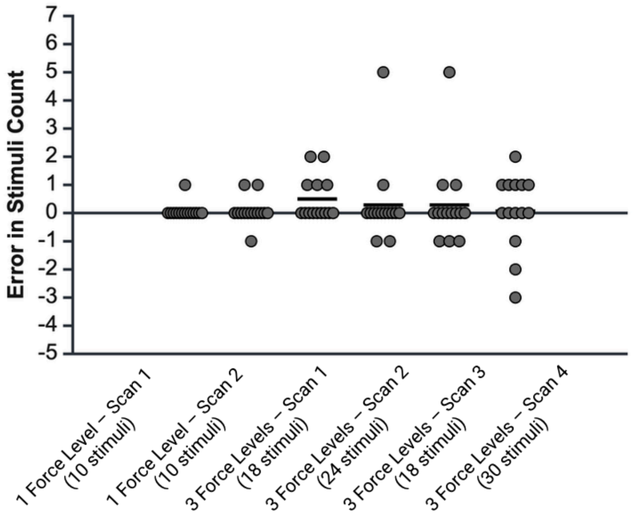

Figure 1: **Errors in Subject's Stimuli Counts:** Scans are presented in sequential order from left to right. Each point represents the error in a subject's reported stimulus count and the true number of stimuli applied.

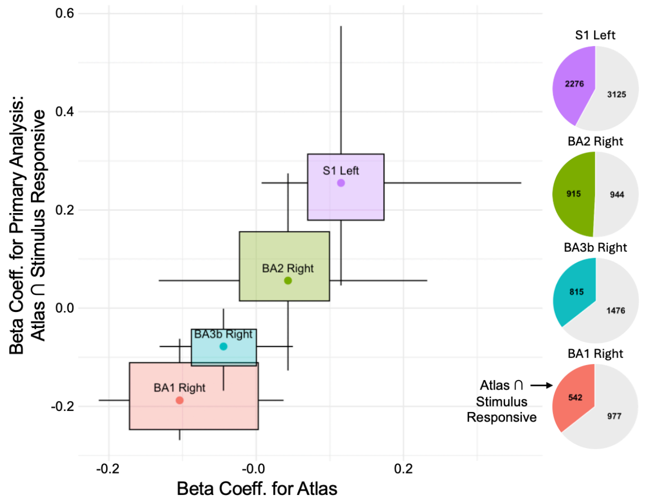

Figure 2: **Evaluating Bias in Primary ROI Definitions:** Box-and-whisker plot examining the beta coefficient between the two approaches for defining our regions of interest within the primary somatosensory cortex. Values represent beta coefficient, and the x and y axes correspond to the two approaches for defining ROIs discussed above. This analysis used stimulus force level as a parametric regressor. The error bars represent the 5th and 95th percentile range. The pie charts on the right show the number of voxels within the ROIs used thought the primary analysis (colored slices) compared to the number of voxels within the atlas-defined region that were not included in our primary analysis (gray slices).

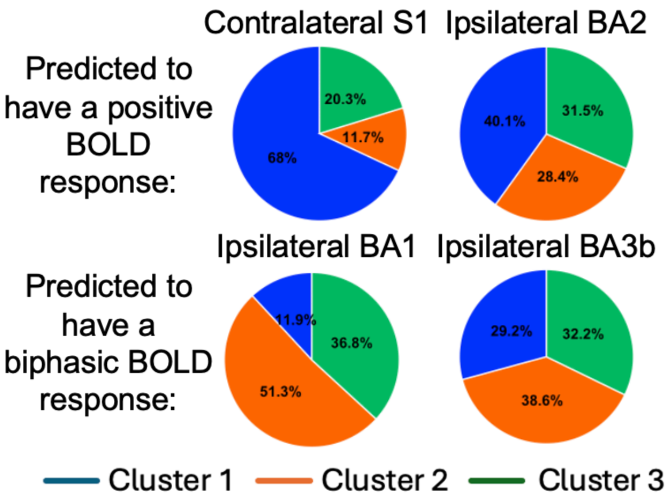

Figure 3: **Full Spatial Organization of AFNI TENT Response Patterns:** Using AFNI's TENT and 3dkmeans functions we found 3 distinct response pattern clusters. Here we compare the spatial organization of each of these response patterns within all regions of interest explored in this work. In both the contralateral S1 and ipsilateral BA2 the positive BOLD response pattern (cluster 1) is most common. In ipsilateral BA1 the biphasic BOLD response pattern (cluster 2) is most common. In ipsilateral BA3b there is approximately equal prevalence of each of the 3 response patterns.

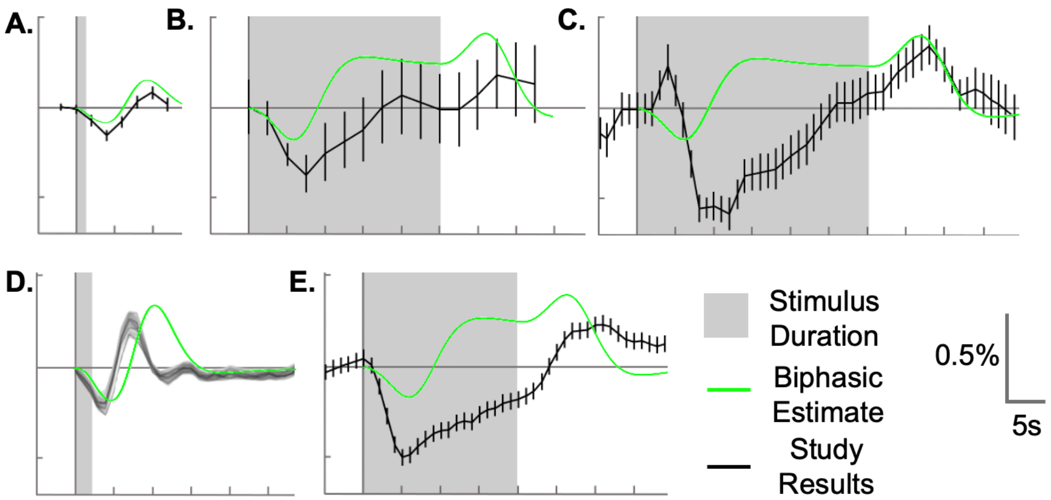

Figure 4: **Qualitative Comparison Across Tactile and Visual Studies:** The TWOGAM HRF was convolved with the stimulus duration of prior studies (green curves in A-E). We qualitatively compared this with fMRI signal changes from several studies (black curves in A-E). The studies include tactile stimulation from **(A)** the current study (Fig. 5C), **(B)** Hlushchuk and Hari (2006, Fig. 2), and **(C)** Kastrup et al. (2008, Fig. 3b). In addition we show responses to visual stimulation from **(D)** de la Rosa et al. (2021, Fig. 2), and **(E)** Shmuel et al. (2006, Fig. 1d). For the de la Rosa study, our HRF was shifted 1.5 s earlier to align with the reported data. The black curves are adapted from the corresponding studies.
